# Our Hidden Enemy: Ultra-Processed Foods, Inflammation, and the Battle for Heart Health

**DOI:** 10.7759/cureus.47484

**Published:** 2023-10-22

**Authors:** Khalid Sawalha, Vyomesh Tripathi, Deya Alkhatib, Luay Alalawi, Ahmed Mahmood, Thomas Alexander

**Affiliations:** 1 Department of Cardiometabolic Medicine, University of Missouri Kansas City School of Medicine, Kansas City, USA; 2 Department of Medicine, University of Missouri Kansas City School of Medicine, Kansas City, USA; 3 Department of Cardiology, Yale School of Medicine, New Haven, USA; 4 Department of Cardiology, Corpus Christi Medical Center Bay Area, Corpus Christi , USA; 5 Department of Cardiology, Corpus Christi Medical Center Bay Area, Corpus Christi, USA

**Keywords:** gut microbiota, chronic inflammation, atherosclerosis, cardiovascular diseases, ultra-processed food

## Abstract

Over the past few decades, we have witnessed unprecedented growth in new data that has fundamentally changed our traditional understanding of the progression of atherosclerotic plaques, as well as our strategies for preventing cardiovascular diseases, especially atherosclerosis. It was once believed that atherosclerosis was primarily caused by abnormal lipid buildup in the vessel intima, leading to plaque growth and luminal stenosis, with or without rupture. This perspective has now evolved to encompass more complex pathways, wherein the accumulation of abnormal products of oxidation and inflammation are the most likely factors mediating the growth of atherosclerotic plaques. The review aims to provide a comprehensive and detailed exploration of the relationship between ultra-processed foods, chronic inflammation, cardiovascular diseases, obesity, insulin resistance, and the role of the gut microbiota. It touches on several important aspects of modern diet and health.

## Introduction and background

What is ultra-processed food, and why is it important

Ultra-processed foods were defined as industrial formulations which, besides salt, sugar, oils, and fats, include substances not used in culinary preparations, in particular additives used to imitate sensorial qualities of minimally processed foods and their culinary preparations [1]. They usually include items such as packaged snacks, frozen meals, and sugary drinks. Ultra-processed foods account for nearly 57.9% of caloric intake and 89.7% of the added sugars consumed in the United States [1].

**Figure 1:**
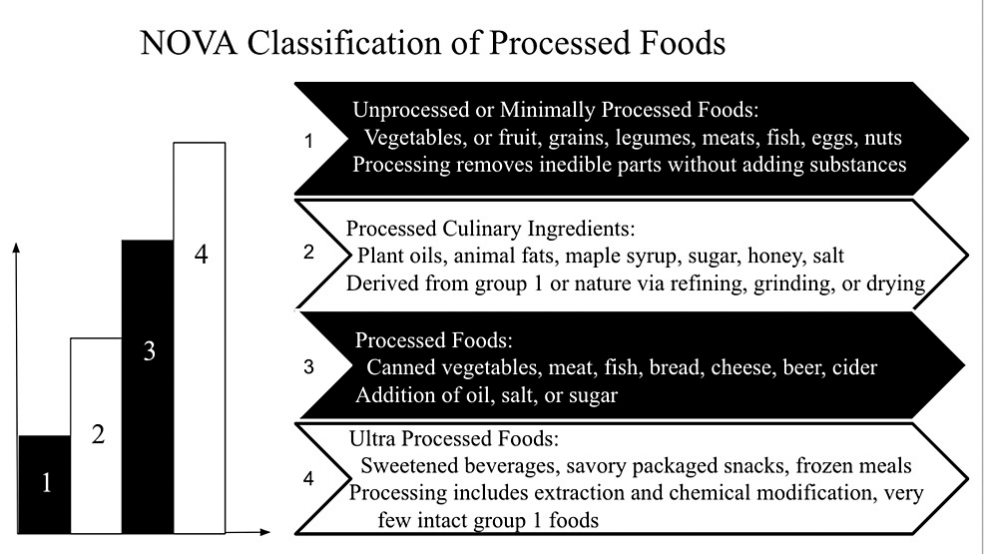
The four main groups of the NOVA classification system for processed foods. Author's own work [5].

In the early 2000s, researchers at the Center for Epidemiological Studies in Health and Nutrition at the University of São Paulo, Brazil, developed a new classification system named the NOVA, which is a framework used to categorize foods and beverages based on the extent and purpose of their processing [2-5]. The system classifies foods into four main groups, including unprocessed or minimally processed foods, processed culinary ingredients, processed foods, and ultra-processed food and drink products (Figure 1) [5]. While the NOVA classification provides a useful framework for understanding the degree of food processing, it's worth noting that it's a general categorization and doesn't specifically label foods as "ultra-processed." Instead, it categorizes foods based on the extent and purpose of their processing, allowing researchers and health professionals to analyze the potential impact of different food types on health and nutrition [5].

Several studies have demonstrated that a diet high in ultra-processed foods is associated with an increased risk of inflammation, a significant factor in the development of chronic diseases such as cardiovascular disease. Srour et al. conducted a study in France involving 105,000 adults who were followed up over a five-year period to investigate various diets. In their research, individuals who had the highest consumption of ultra-processed foods exhibited a 28% greater risk of developing cardiovascular disease compared to those with the lowest consumption [6]. Furthermore, the researchers observed that these individuals showed higher levels of inflammatory markers in their serum, consistent with the chronic inflammation that promotes cardiovascular disease [6].

Another prospective study, involving over 44,000 adults, demonstrated a positive association between the consumption of ultra-processed foods and all-cause mortality, especially in the context of cardiovascular issues [7]. This was also supported by another study published by Jull et al. suggesting that higher consumption of ultra-processed foods, was associated with a greater cardiovascular disease incidence and mortality. This risk was also noted with each additional serving, leading to a 9% increased risk in CVD mortality [8].

**Figure 2:**
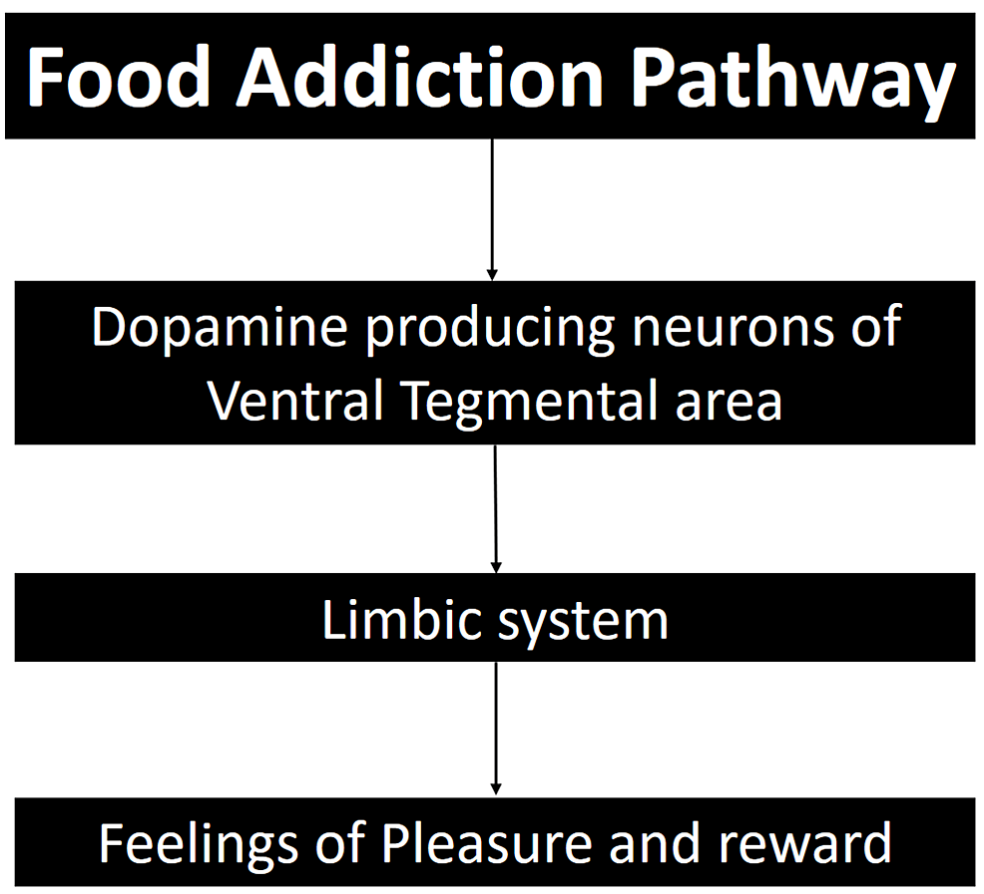
The shared pathway between food and substance addiction. Author's own work [9].

The effects of ultra-processed foods extend beyond the heart to impact brain pathways and foster addiction. According to a study conducted by the University of Michigan, which utilized the Yale Food Addiction Scale, food addiction and substance use disorders exhibited similar patterns in the activation of brain reward circuitry (Figure 2) [9]. This suggests that ultra-processed foods can not only result in long-term detrimental effects on human health but also present challenges to recovery.

## Review

Over the past five decades, the food industry has witnessed a major transition away from saturated fats over concerns of developing coronary heart disease to substitute it with sugars that have become increasingly prevalent in diets, especially fructose [10]. Fructose consumption has gone from ~37g in the 1970s to ~55g per day by the early 2000s [11]. This lines up with the increased global burden of cardiometabolic diseases, primarily hypertension, insulin resistance, dyslipidemia, and central obesity. Despite all the significant efforts made to advance our current understanding of the pathophysiological differences and clinical implications between these disease clusters, more needs to be done to tackle these burdens.

So, what do we know regarding UPF and its link to cardiometabolic diseases? Not that much. However, we know that coronary heart disease, particularly atherosclerosis, is a major cause of disability and death among patients with cardiometabolic diseases because of coronary, cerebrovascular, and peripheral arterial disease [12]. Furthermore, significant endothelial, vascular smooth muscle cell, and platelet function abnormalities as well as increased production of several prothrombotic factors are present in diabetes mellitus [12]. Additionally, hyperglycemia, increased free fatty acids, insulin resistance, decreased nitric oxide bioavailability, and increased oxidative stress disrupt intracellular signal transduction and activate receptors for advanced glycation end products. This state promotes chronic inflammation that contributes to cardiovascular disease [6-12].

Even when the concerns raised in the 2003 American Heart Association publication linking metabolic abnormalities with the development of CVD in adults in Westernized societies were accepted, similar concerns addressed in children, adolescents, and young adults never gained support or scientific traction [13]. The theory of atherosclerosis has evolved from accumulation of lipid-laden macrophages and proliferation of smooth muscle cells in coronary arteries to a complex interplay of different pathways driven by chronic levels of inflammation [14]. Consequently, is it time to unify our approach and concentrate on when this chronic inflammation truly starts? What causes it? Can We reverse it? An interesting perspective to consider is the obese state.

Inflammatory processes and gut microbiota

**Figure 3:**
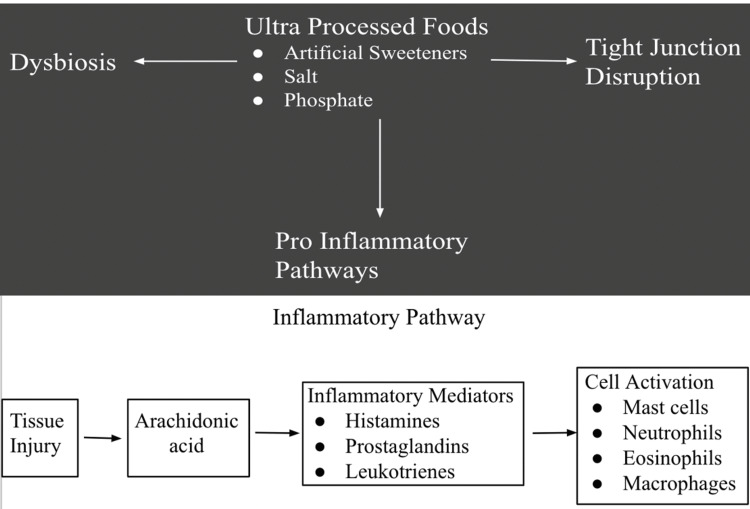
The inflammatory pathways in which ultra-processed foods affect our bodies. Author's own work [15].

Obesity is well known to be characterized by a state of chronic low-grade inflammation [15]. This low-grade inflammation may be attributed to the repeated exposure of high-fat and high-carbohydrate diets, inducing chronic metabolic endotoxemia. This persistent activation of the immune system results in the proliferation of proinflammatory mediators and the alteration of glucose metabolism (Figure 3) [15]. Predictably, this is followed by hyperglycemia when the body can no longer produce sufficient insulin to manage the demand of the glucose load. Moreover, mankind entered a phase in our evolution where food is abundantly available and palatable, leading to a continuous struggle against the health implications of the foods we consume.

Consider that consuming a sausage, egg, and cheese sandwich increases endotoxemia by 40% and leads to persistent immune activation for at least six hours, whereas opting for healthier meal choices, such as oatmeal, orange juice, and fruit, results in a 10% increase in endotoxemia or immune-inflammatory responses [16]. 

Obesity and insulin resistance contribute to chronic inflammation. This process begins with cellular-level glycosylation, which is the most common post-translational protein modification, disrupting interactions between cells and molecules [17,18]. This is one of the reasons why we see mitochondrial dysfunction with the release of mitochondria-derived damage-associated molecular patterns (DAMPs) [18]. Mitochondrial DAMPs have recently become the subject of intensive research due to their possible involvement in conditions associated with inflammation by interacting with receptors similar to those involved in pathogen-associated responses, such as aging and degenerative diseases [17,19]. This inflammatory process also induces oxidative stress and reduces cellular antioxidant capacity, causing an overproduction of free radicals that permanently impair fatty acids and proteins of the cell membrane [20]. In addition, chronic inflammation seems to hinder the body’s ability to produce autophagosomes that normally clean up cell debris and damaged cells. The lack of autophagy then contributes to neurodegenerative diseases. In addition, chronic inflammation is linked with DNA methylation of macrophage genes inhibiting the body's natural defense system [21].

Coming to the role of the human gut microbiota in insulin resistance and obesity, data from mice studies have shown that germ-free mice on a high-fat diet gain less weight than control mice on the same diet, and they were also protected from insulin resistance [22]. The exact mechanisms of protection from insulin resistance are not well understood yet. However, we do know that AMPK activity was enhanced in the muscle and liver of germ-free mice, which played a huge role in weight loss by the process of enhanced fatty acid oxidation and energy expenditure [22]. Additionally, germ-free mice that were infected with gut microbiota of conventionally raised mice displayed an increase in both body weight and fat content associated with insulin resistance and glucose intolerance [23]. Furthermore, transplanting intestinal microbiota from wild-type to germ-free mice enhanced the adiposity associated with insulin resistance [24]. These studies seem to indicate that there is a cause-effect relationship between the gut microbiota, fat content, and insulin resistance.

In addition to insulin resistance and obesity, disruptions to gut microbiota can lead to neurodegenerative changes. Microbiota produce short-chain fatty acids (SCFAs) that act as signaling molecules in the central nervous system [25,26] an essential function that has allowed the gut microbiota to become recognized as its own metabolic organ [25]. Dysbiosis as a process represents decreased numbers of bacteria that produce SCFAs and an increase in harmful microorganisms with lipopolysaccharide (LPS) endotoxins [27]. LPS activates immune cells, which release proinflammatory cytokines (IL-1a, IL-1b, TNF-a, and IL-6) that cross the blood-brain barrier and bind microglial receptors, leading to the onset of neuroinflammation [28].

Neuroendocrine and immunologic cells can also be affected, causing dysregulation of neurotransmitters and resulting in the different clinical manifestations that characterize NDs [29]. High-fat diets also directly cause neurodegenerative disease processes, evidenced by several studies in animal models showing how high-fat diets affect microglial function [30], cause cognitive deterioration and neuroinflammation [31], and play a role in the development of Alzheimer's disease [32]. In addition, a cross-sectional study conducted by the National Health and Nutrition Examination Survey (NHANES) that involved 3632 participants over 60 years old found that consuming UPF was inversely related to performance in the Animal Fluency test, which assesses language and executive function [33]. This was also supported by a study conducted by Finnish researchers that explored the link between the Desulfovibrio (DSV) bacteria and Parkinson's disease (PD). PD is caused when LPS produces local inflammation and oxidative stress in the intestine, thus initiating the a-synuclein (aSyn) deposition that disseminates to the CNS. Pathologic aSyn can lead to neuronal death, microglial activation, and subsequent activation of TLRs, triggering a vicious cycle of neuroinflammation [33]. The Finnish study demonstrated that worms fed DSV bacteria from PD patients had both more and larger alpha-syn aggregates than worms fed DSV bacteria from healthy individuals or E. coli [34]. Worm-fed DSV bacteria also had higher mortality, especially in patient strains, possibly due to the high volume of alpha-syn aggregates and bacterial toxicity [34].

Possible future directions of ultra-processed food

So, what can be done to try to improve UPF? We can start by looking at the results of a complex study conducted by a Scientific Advisory Team who worked with the Kuwaiti Danish Dairy Company to conduct an evaluation of their entire commercial food and beverage portfolio. The authors developed a tiered “Metabolic Matrix” founded on three science-based principles: (1) feed the gut, (2) protect the liver, and (3) support the brain [35].

The first pillar is to “feed the gut” quality fiber which has been shown to improve glycemic control [36], as it contributes to the production of SFCA that provides nourishment for colonic bacteria and serves as an anti-inflammatory agent [37]. SFCAs also have downstream effects of keeping the integrity of the gut barrier and improving glucose and lipid metabolism, which improves cardiovascular risk [38-40].

The second pillar is to “protect the liver” as toxins in food can make the liver dysfunctional, leading to hepatic insulin resistance and hyperinsulinemia, which drive aberrant cellular growth and chronic metabolic disease [41]. These compounds are highly abundant in ultra-processed foods [42], so reduction in substrate exposure is a logical preventative measure.

The third pillar is to “support the brain” as the omega-6 to omega-3 ratio consistent with Western-type dietary patterns appears to be a primary contributing factor to the premature development of heart disease and stroke and neurodegenerative diseases [43-45]. An improved selection of ingredients to substitute inflammatory omega-6 fatty acids for brain-essential omega-3 fats could go a long way to preventing neurodegenerative changes, heart disease, and stroke [46,47].

It is clear by now that high fat-high carbohydrate food can interact with the innate immune system and set the inflammatory environment of the body. Keep that exposure constant and you have a chronic state. We know that limiting these foods and altering the pathway of food exposure through the gastrointestinal tract by means of gastric bypass reverse endotoxemia and the persistent activation of the immune system [48]. Moreover, this procedure resolves the chronic inflammatory state and restores proper insulin signaling to resolve the diabetic state. Insulin resistance is the natural outcome of persistent activation of the immune system and consequently a key factor in the development of atherosclerosis. Modifying the nutrient supply (intake) and the frequency of interactions with the immune system may reduce or even reverse the cardiometabolic abnormalities.

## Conclusions

Given the social resistance of giving up fast food, re-engineering ultra-processed food to become healthier might be the possible solution for this growing pandemic based on the current available results seen in the Kuwaiti Danish diary above. Despite our best efforts, therapies and interventions have limited efficacy and outcomes remain basically unchanged. Perhaps, it is then time to start addressing them during childhood, adolescence, and in young adults. This might prove to be our most enduring and effective legacy.
